# Quantitative Ultrasound for Hepatic Steatosis: A Systematic Review Highlighting the Diagnostic Performance of Ultrasound-Derived Fat Fraction

**DOI:** 10.3390/diagnostics15202640

**Published:** 2025-10-20

**Authors:** Dimitrios Kavvadas, Vasileios Rafailidis, Aris Liakos, Emmanouil Sinakos, Sasan Partovi, Theodora Papamitsou, Panos Prassopoulos

**Affiliations:** 1 Department of Clinical Radiology, AHEPA University Hospital, Aristotle University of Thessaloniki, 54636 Thessaloniki, Greece; vrafaili@auth.gr (V.R.); pprasopo@auth.gr (P.P.); 2 Clinical Research and Evidence-Based Medicine Unit, Second Medical Department, Aristotle University of Thessaloniki, 54642 Thessaloniki, Greece; arliakos@auth.gr; 3 Fourth Medical Department, Aristotle University of Thessaloniki, 54642 Thessaloniki, Greece; esinakos@auth.gr; 4 Interventional Radiology, Cleveland Clinic Main Campus, Cleveland, OH 44195, USA; sxp509@case.edu; 5 Laboratory of Histology-Embryology, School of Medicine, Faculty of Health Sciences, Aristotle University of Thessaloniki, 54124 Thessaloniki, Greece; thpapami@auth.gr

**Keywords:** ultrasound, UDFF, MRI-PDFF, liver steatosis, MASLD

## Abstract

**Background/Objectives**: Metabolic-dysfunction-associated steatotic liver disease (MASLD) is a leading cause of chronic liver disease worldwide, requiring accurate and accessible diagnostic tools. **Methods**: A systematic review evaluated the diagnostic performance of Ultrasound-Derived Fat Fraction (UDFF), with a primary focus on prospective studies comparing UDFF to MRI Proton Density Fat Fraction (MRI-PDFF) as the reference standard and a secondary appraisal of its performance against other modalities. Additional parameters, such as technical feasibility, inter-observer agreement, and proposed thresholds, were summarized to support clinical applicability. **Results**: Seven prospective MRI-based studies (*n* = 862) demonstrated excellent correlation (average r = 0.848) and reproducibility (inter-observer intraclass correlation coefficient ICC = 0.978, intra-observer ICC = 0.980) of UDFF, with high diagnostic accuracy across steatosis grades (AUCs ≥ 0.89). Additional studies comparing UDFF with Controlled Attenuation Parameter (CAP), histology, and other quantitative ultrasound techniques (attenuation- or backscatter-based methods) confirmed high sensitivity and specificity, particularly for advanced steatosis, and emphasized the potential of UDFF as a comprehensive quantitative biomarker. Proposed UDFF cut-offs for mild, moderate, and severe steatosis ranged from 5% to 23%, demonstrating high sensitivity and specificity. Factors like body position, probe pressure, and visceral fat influenced measurements, underscoring the need for standardized protocols. **Conclusions**: UDFF seems to offer a reliable and cost-effective quantitative ultrasound modality. So far, it correlates strongly with MRI-PDFF and accurately grades steatosis, especially for S2–S3. Given cut-off variability and protocol sensitivity, broad routine adoption may be premature. Therefore, we recommend further studies focusing on standardized acquisition and cut-off calibration to MRI-PDFF.

## 1. Introduction

Metabolic-dysfunction-associated steatotic liver disease is currently the most common cause of chronic liver disease worldwide, with an estimated global prevalence of up to 38% in the general population [1,2,3,4,5]. The term MASLD encompasses a broad clinical spectrum of hepatic steatosis, ranging from simple fatty infiltration, which typically does not cause symptoms or complications, to metabolic-dysfunction-associated steatohepatitis (MASH), which is characterized by inflammation and fibrosis [6].

The Proton Density Fat Fraction (PDFF) sequence in magnetic resonance imaging (MRI) is considered the “gold standard” for the non-invasive quantification of liver fat due to its high accuracy and ability to assess the entire organ [3]. MRI-PDFF measures the fat fraction by measuring the total proton content of the tissue, providing reliable results for the assessment and staging of steatosis [7]. Despite its advantages, MRI-PDFF is associated with high costs, limited availability, and contraindications to metallic implants. Additionally, its use is limited in routine monitoring due to the requirement for specialized equipment and trained personnel [3].

B-mode ultrasound is the most widely used imaging method for the non-invasive assessment of hepatic steatosis [8]. It is based on the subjective observation of liver echogenicity, with increased fat content making the organ appear more echogenic [8]. Although it is a fast and non-invasive technique, it has limitations in detecting mild steatosis, it is operator-dependent, and it provides qualitative rather than quantitative assessments [4,5]. Specifically, B-mode ultrasound qualitatively detects hepatic fat infiltration with high sensitivity when it exceeds 20% (increased liver brightness, vessel blurring, acoustic wave attenuation, focal fatty infiltration), meaning that liver fat content ranging from 5% to 20% may go undiagnosed [8] Jamalinia et al. found that even mild hepatic steatosis is associated with increased cardiovascular disease risk [9]. Similarly, Gao et al. linked the risk of chronic kidney disease in patients with MASLD to the severity of hepatic steatosis, starting from mild steatosis [10].

**Figure 1:**
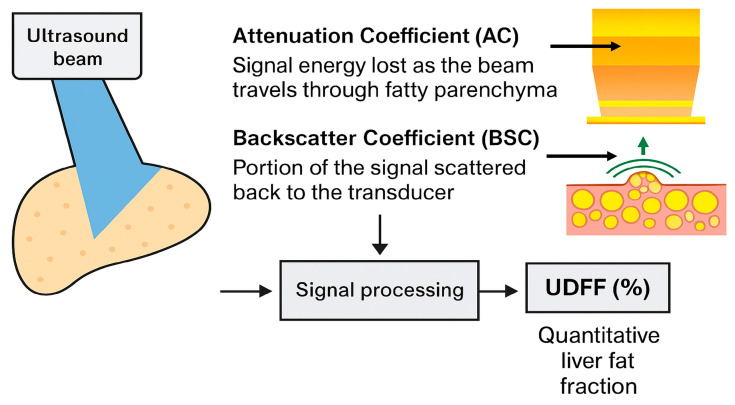
Mechanistic illustration of UDFF. Increased fat in steatotic liver results in higher AC values and stronger BSC signals. UDFF integrates these parameters through signal processing to provide a quantitative percentage of hepatic fat content.

The increasing demand for non-invasive and more accurate methods for assessing hepatic steatosis, combined with the widespread use of ultrasound, has led to the development of new techniques, such as Ultrasound-Derived Fat Fraction (UDFF). UDFF utilizes the combination of two parameters, the attenuation coefficient (AC) and the backscatter coefficient (BSC), to estimate liver fat content with greater accuracy compared to conventional echogenicity assessment [3,4,5,11]. More specifically, UDFF applies radiofrequency (RF) waves that reflect off of liver tissue to assess the attenuation and backscatter characteristics of hepatic fat (Figure 1) [3,4,5].

The aim of this review is to summarize the existing literature for the diagnostic performance of UDFF in assessing hepatic steatosis using MRI-PDFF as the reference standard and comparing UDFF with other parameters, as well. This study aims to determine, based on the existing literature, whether UDFF can serve as a reliable, cost-effective, and accessible modality for routine clinical evaluation of hepatic steatosis in adults with suspected or confirmed MASLD.

## 2. Materials and Methods

A systematic literature search was conducted in PubMed, Scopus, Embase, Web of Science, and the Cochrane Library, covering studies published from January 2014 to April 2025. The search included the following generic keywords: “Ultrasound-Derived Fat Fraction (UDFF)”, “Quantitative ultrasound”, “MRI-PDFF”, “Hepatic steatosis”, and “Liver fat quantification”. Boolean operators (AND, OR) and Medical Subject Headings (MeSH) were applied where applicable.

The main question regarding the diagnostic performance of UDFF with MRI-PDFF as a reference was based on prospective studies assessing UDFF against MRI-PDFF in adult patients that were included in the abovementioned analysis. Not all parameters (e.g., AUC, reproducibility) were uniformly reported across studies. Two independent reviewers screened titles, abstracts, and full texts using the QUADAS-2 tool, covering four domains: patient selection, index test, reference standard, and flow/timing. Any disagreements were resolved through discussion and consensus. A third reviewer was consulted in cases of unresolved disagreement (Appendix A).

Although the primary focus was diagnostic performance against MRI-PDFF, additional parameters, such as technical feasibility, inter-observer agreement, and proposed thresholds, were summarized to support clinical applicability. Studies that used CAP or liver biopsy as the reference standard were analyzed separately from the first analysis to assess the cut-offs and add further insights regarding the diagnostic performance of UDFF.

For the main question, studies were included if they met the following criteria:

✓ Adult population.
✓ UDFF assessment of hepatic steatosis.
✓ MRI-PDFF as a reference standard.
✓ Quantification of liver fat content.

Exclusion criteria were as follows:

○ Studies involving a pediatric population.
○ Review articles without explicit UDFF evaluation.

Titles and abstracts were screened for relevance, followed by full-text assessment for eligibility using the QUADAS-2 tool. From the eligible studies, the following data were extracted:

⮚ Study characteristics (authors, year, study design).
⮚ Sample size and population demographics.
⮚ Imaging techniques used (UDFF and MRI-PDFF acquisition details).
⮚ Main findings, including AUC, ICC values, and diagnostic performance metrics.

A detailed framework to compare UDFF against MRI-PDFF across studies was designed as follows:

⮚ Reference definition: Steatosis on MRI-PDFF was defined as above 5.0% or 5.5%, according to each study’s protocol [7,12].
⮚ MRI technique harmonization: Field strength (1.5T vs. 3T), vendor/sequence (multi-echo Dixon implementation), number of echoes, T2* correction, fat-spectral model, and reconstruction method [7,12].
⮚ ROI comparability: Lobe/segment measured and ROI depth [6,13,14,15].
⮚ Timing and preparation: Interval between UDFF and MRI (days), fasting status, body position, and breath-hold [6,13,14,15].
UDFF acquisition: Transducer, frequency, measurement count, quality metrics, and any protocol deviations from Siemens UDFF guidance [11,16].
⮚ Key diagnostic parameters: Correlation (r), agreement (ICC), diagnostic accuracy (AUC, Se/Sp per steatosis grade), with 95% CIs when reported [4,5,17,18].

Key diagnostic parameters were as follows: [4,5,17,18]:

⮚ AUC: Measure of diagnostic accuracy. Values range from 0.5 (no discrimination) to 1.0 (perfect discrimination).
⮚ ICC: Reliability statistics describing inter- or intra-observer agreement. Values >0.90 indicate excellent agreement.
⮚ UDFF cut-off values: Thresholds (expressed as %) used to define mild, moderate, and severe hepatic steatosis.
⮚ Sensitivity and specificity: The true positives and true negatives correctly identified by UDFF at a given threshold.
⮚ r Pearson’s correlation: Between UDFF and MRI-PDFF values. Values close to +1 indicate strong positive correlation.

**Figure 2:**
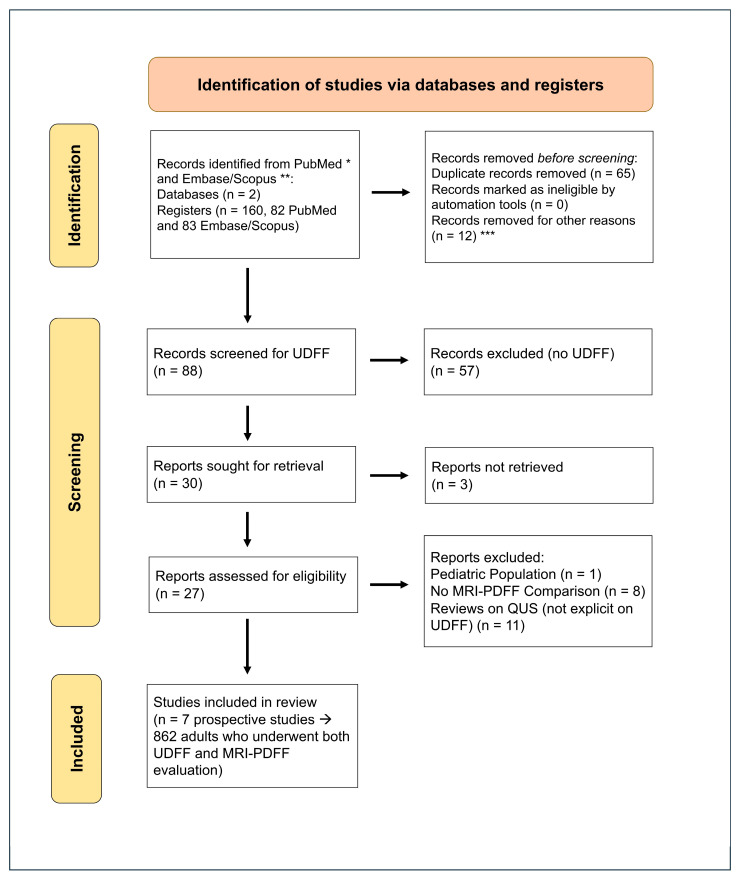
PRISMA guidelines for assessing the diagnostic performance of UDFF vs. MRI-PDFF (source: Page MJ, et al. [19]). * “Ultrasound-Derived Fat Fraction” OR “UDFF” OR “quantitative ultrasound” OR “QUS” OR “attenuation coefficient” OR “backscatter coefficient”) AND (“Proton Density Fat Fraction” OR “MRI-PDFF” OR “magnetic resonance imaging PDFF” OR “MRI liver fat quantification”). ** TITLE-ABS-KEY(“Ultrasound-Derived Fat Fraction” OR “UDFF” OR “quantitative ultrasound” OR “QUS” OR “attenuation coefficient” OR “backscatter coefficient”) AND TITLE-ABS-KEY(“Proton Density Fat Fraction” OR “MRI-PDFF” OR “magnetic resonance imaging PDFF” OR “MRI liver fat quantification”) AND TITLE-ABS-KEY(“liver”). *** Records excluded before the first UDFF publication: Labyed et al. (2020) [11].

The study selection process followed the PRISMA guidelines (Figure 2, source: Page MJ, et al. [19]), and the review was registered with INPLASY [20].

### Statistical Analysis

When at least three comparable studies were available for the UDFF vs. MRI-PDFF analysis, we performed random effects meta-analyses with inverse-variance weighting. For correlation coefficients (r), we used the Fisher z-transform, pooled on the z scale (τ^2^) and back-transformed to r; we report 95% CIs, I^2^, Q, τ^2^, and 95% prediction intervals. Inter- and intra-observer ICCs were analyzed using the same Fisher-z approach when sample sizes were available. For each metric, studies were pooled only when they reported a compatible effect measure with sufficient information to compute inverse-variance weights (e.g., study-level r with n or ICC with CI). Studies lacking such data were synthesized narratively and, where applicable, included in other quantitative summaries (e.g., ICC).

## 3. Results

### 3.1. Study Selection and Characteristics

A total of seven prospective studies were included regarding UDFF vs. MRI-PDFF performance, with a total of 862 patients. The recruited patients were of varying demographic and clinical profiles, focused on adult patients, with mean ages ranging from mid-40s to early 70s and a balanced gender distribution. Most of the study populations included individuals with known or suspected MASLD with or without comorbidities, such as type 2 diabetes mellitus, obesity, dyslipidemia, or hypertension. While all studies included patients with metabolic risk factors, some studies enrolled broader populations undergoing liver evaluation, and MASLD-specific stratification was not explicitly confirmed in all cases.

### 3.2. Diagnostic Performance of UDFF vs. MRI-PDFF

UDFF has been compared to MRI-PDFF, demonstrating high accuracy and reproducibility in the diagnosis and classification of hepatic steatosis [21]. A total of seven prospective studies were found based on the PRISMA reporting guidelines, including a direct comparison of UDFF to MRI-PDFF as a reference method (Table 1). Labyed et al. also included liver biopsy and found that UDFF demonstrated a high level of agreement with both liver biopsy and MRI-PDFF [11]. This was the first study to present UDFF, and it eliminated the need to scan phantoms in a clinical setting. A similar dataset was reported by Dillman et al., who presented waist circumference as a significant independent predictor of UDFF. They also recommended that three UDFF measurements may be obtained in clinical practice, instead of five [13]. However, increased variability with greater steatosis and a potential bias toward higher UDFF values were reported in another study [14]. In addition, the potential for further variability in UDFF measurements is highlighted by Qi et al. due to changes in body position and probe pressure [6]. Song et al. provide more data on this subject, concluding that end-expiratory, supine, and fasting images had the lowest variability [22]. Wang et al. note that there are limited data on UDFF in cirrhotic patients and severe steatosis cases [15]. Nevertheless, the abovementioned studies reported a strong average correlation between UDFF and MRI-PDFF measurements (ICC: 0.843; r: 0.848).

Agreement/reproducibility for UDFF was excellent (inter-observer ICC ~0.9–0.99; intra-observer ICC ~0.96–0.99). By steatosis grade, diagnostic accuracy (AUC) was as follows:

- S1 (mild): 0.747–0.99 (heterogeneous at MRI-PDFF threshold of 5.0% and in healthy/low-fat cohorts).
- S2 (moderate): 0.95–0.96 (consistently excellent).
- S3 (severe): 0.95–0.97 (consistently excellent).

When stratified by MRI-PDFF threshold, studies using a threshold above 5.5% showed slightly higher AUCs at S1 than those using a threshold above 5.0%, consistent with expected threshold effects (Table 1). Other studies also calculated these ICCs and found similar results [2,18].

#### Correlation Analysis of UDFF vs. MRI-PDFF

⮚ Five studies (*n* = 570: Labyed 2020 [11]; Dillman 2022 [13]; De Robertis 2023 [3]; Qi 2024 [6]; Wang 2024 [15]) were pooled using a random effects model on the Fisher-z scale. The pooled correlation was r = 0.85 (95% CI 0.81–0.89), with I2 = 67%, Q = 12.19, and τ^2^ = 0.019; the prediction interval was 0.74–0.92. This supports a strong association between UDFF and MRI-PDFF while indicating moderate between-study heterogeneity.
⮚ Inter-observer ICC (four studies; *n* = 583: Kubale 2024 [14]; Qi 2024 [6]; Song 2024 [22]; Wang 2024 [15]): pooled ICC = 0.95 (95% CI 0.935–0.957), I^2^ = 40%, Q = 5.04, and τ^2^ = 0.0049.
⮚ Intra-observer ICC (four studies; n = 524: Dillman 2022 [13]; Kubale 2024 [14]; Qi 2024 [6]; Song 2024 [22]): pooled ICC = 0.98 (95% CI 0.966–0.991), I^2^ = 93%, Q = 43.54, and τ^2^ = 0.112.
⮚ Thresholds were provided based on descriptive statistics:
  ○ S1: Median 0.90, range 0.747–0.99 (n-weighted mean ~0.874).
  ○ S2: Median 0.95, range 0.950–0.960 (n-weighted mean ~0.952).
  ○ S3: Median 0.95, range 0.950–0.970 (n-weighted mean ~0.955).

Variability at S1 aligns with differences in MRI-PDFF threshold (≥5.0% vs. ≥5.5%), ROI matching, and acquisition factors.

### 3.3. Diagnostic Performance of UDFF vs. Other Modalities

UDFF has been compared to other ultrasound modalities across multiple studies (Table 2). Gao et al. demonstrated good reproducibility of both UDFF and auto-point Shear Wave Elastography (pSWE) in adults, highlighting the importance of simultaneously assessing hepatic steatosis and fibrosis [2,23]. Sporea et al. reported a good correlation between UDFF and Controlled Attenuation Parameter (CAP) and increased UDFF specificity in severe steatosis cases [24]. Nakamura et al. reported excellent agreement between UDFF and histological fat assessment [21]. There was a good correlation between UDFF values and the degree of steatosis in known MASLD cases [14,25,26], with consistency across liver segments [25]. UDFF values were positively correlated with the CAP score and the Hamaguchi score [14,25,26].

### 3.4. Proposed Cut-Off Values for UDFF in Steatosis Grading

UDFF has an excellent average AUC for S1 steatosis (0.893 for a PDFF cut-off > 5%) and excellent inter-observer agreement (ICC: 0.947) and intra-observer agreement (ICC: 0.979). So far, UDFF cut-off values of 5.5%, 15.5%, and 17.5% have been proposed, demonstrating high sensitivity and specificity for detecting mild, moderate, and severe hepatic steatosis, respectively (Table 3) [6].

These cut-offs (5.5%, 15.5%, and 17.5%) were chosen as they represent the most frequently validated thresholds across multiple independent studies, especially those using MRI-PDFF as the reference standard (e.g., Qi et al. [6], Sporea et al. [24]). Furthermore, their diagnostic performance (AUCs > 0.90, Se/Sp > 0.85) was among the highest reported, supporting their clinical utility in steatosis stratification.

Nakamura et al. used liver biopsy as the gold standard and found excellent diagnostic performance of UDFF across all steatosis grades, with sensitivity exceeding 90% for S1 steatosis [21]. Compared to CAP, UDFF showed excellent specificity, especially for moderate and severe steatosis (cut-offs > 10% and >15%) [24]. In studies using MRI-PDFF, UDFF achieved high AUC values (≥0.90), particularly for higher steatosis grades [3,6,11,13,14,15,27].

### 3.5. Factors Affecting UDFF Performance

Metabolic factors, such as blood triglyceride levels, pyruvate transaminase levels, and visceral fat, may influence UDFF measurements [14,25,26]. Although higher UDFF values were moderately correlated with increased BMI, BMI itself was not an independent determinant of UDFF’s diagnostic accuracy [13,26]. Waist circumference, however, was independently associated with improved diagnostic performance, indicating a stronger link to fat distribution relevant to UDFF measurements [13]. Similarly, visceral fat area was strongly associated with higher UDFF values [22,26]. Food intake did not appear to affect the accuracy of UDFF, suggesting for certain studies that fasting is unnecessary for liver fat quantification. However, it is important to emphasize that fasting is required when assessing liver stiffness for fibrosis detection [5,28]. Other studies performed 6 h fasting [22]. Nevertheless, fasting state did not significantly affect obtained values.

During the performance of the examination, supine position was more reliable in comparison to lateral decubitus due to rib shadows [22]. Respiration affects stability, and therefore end-expiratory breath-hold is preferred for more consistent readings [22]. Based on recent reviews, thresholds for detecting and grading liver steatosis might vary depending on the ROI’s depth [29]. Skin-to-Capsule distance (StCd) affected the signal in obese patients, independently [22].

Summing up the technical details of performing UDFF, the majority of these studies followed the technical guidelines provided by Siemens [16]. There were a few modifications, which are presented below, alongside the official guidelines:

○ Patient Preparation: Patient should fast for at least 4 h prior to the examination [4,16].
  - Most studies suggest 6 h [2,3,18,25] of fasting or not at all [14,28].
○ Patient Positioning: Supine or slightly (30°) left lateral decubitus position and right arm raised above their head to optimize intercostal access [4,16].
  - A study also suggests dorsal decubitus position with maximal right arm abduction [25].
○ Transducer Positioning: Perpendicular (90°) to the skin’s surface with plenty of ultrasound gel to ensure proper acoustic coupling [4,16].
○ Selecting Region of Interest (ROI): Artifact-free area within the right lobe of the liver (no vessels, no large hepatic ducts, no rib shadows, etc.) with the ROI at 1.5 to 2 cm below the liver capsule and the liver capsule marker parallel with the echogenic interface of the liver capsule [4,16].
  - A study suggests investigating how the depth at which measurements are made (1.5, 2, 3, 4, and 5 cm below the liver capsule) affects the accuracy and reliability of the UDFF values [30].
○ Patient Breathing Instructions: Before measurement acquisition, ask the patient to hold their breath for 10–15 s until acquisition is complete [4,11,16].
  - Song et al. preferred the end-expiratory breath-hold for more consistent readings [22].
○ Acquisition Procedure: Five separate UDFF measurement samples for each complete examination [4,16].
  - Ten ROIs were suggested to be placed at different levels in the right hepatic lobe, and the median was calculated for analysis [3].

## 4. Discussion

MASLD poses major diagnostic challenges due to the invasive nature of liver biopsy and practical limitations associated with MRI-PDFF [31]. Almost 50% of cases with MASLD may go undiagnosed due to the inability of B-mode to detect liver disease at percentages of 5 to 20%, which accounts for mild and moderate liver steatosis [25]. UDFF is based on combined data from the AC and the BSC, delivering an alternative for assessing hepatic steatosis non-invasively [12,14,15].

The AC indicates the proportion of wave energy lost as it penetrates the liver parenchyma. High intrahepatic fat accumulation will result in greater attenuation of the ultrasound beam [32]. GE Healthcare with LOGIQ E9 has launched the Ultrasound-Guided Attenuation Parameter (UGAP, GE Healthcare, Chicago, IL, USA) modality based on AC, with an excellent AUC for detecting liver steatosis. More specifically, nine studies on UGAP provide an excellent average AUC (0.935, std. 0.026) and a strong correlation with MRI-PDFF (0.792, std 0.072) for detecting liver steatosis > 5% based on MRI-PDFF as a standard [12,33,34,35,36,37,38,39]. Tissue Attenuation Imaging (TAI) is another technique that performs AC “mapping” across a selected region of the liver. It takes advantage of the depth-dependent frequency shifts in the ultrasound signal to assess fat content. This modality is provided by Samsung RS85 (Samsung Medison, Seoul, Republic of Korea), and there are five prospective studies with an excellent AUC (0.908, std. 0.035) and a strong correlation with MRI-PDFF (0.838, std. 0.073) for detecting liver steatosis [40,41,42,43,44]. Canon Applio i800 (Canon Medical Systems, Otawara, Tochigi, Japan) also provides an excellent average AUC for AC (0.911, std. 0043) based on prospective studies [2,23,30,34,45,46]. The correlation with MRI-PDFF was strong (0.731, std. 0.172) for detecting liver steatosis > 6% based on MRI-PDFF as a standard [2,23,30,34,45,46]. Fujifilm ARIETTA 850 (Fujifilm Healthcare, Tokyo, Japan) uses the integrated Attenuation Technique (iATT), a variant of the attenuation measurement function that typically offers automated assessment of the AC from B-mode images, providing a very good AUC (0.873, std. 0.026) and a strong correlation with MRI-PDFF (0.765, std 0.038) for detecting liver steatosis [47,48].

The BSC measures the proportion of wave energy that returns to the transducer after interacting with tissues of varying echogenicity [6]. Backscatter phenomena are based on the density difference between fat and water [17]. Lipid cells have lower density than the water-based cells of the liver parenchyma and, therefore, the speed of sound is lower during the propagation of these specific cells, resulting in higher BSC of fatty liver [17]. A technique that analyzes the statistical distribution of the backscattered ultrasound signal is Tissue Scatter Distribution Imaging (TSI), which is provided by Samsung RS85 (Samsung Medison, Seoul, Republic of Korea). There are six studies providing an excellent AUC (0.914, std. 0.049) and a moderate correlation with MRI-PDFF (0.634, std. 0.341) for detecting liver steatosis [40,41,42,43,44,49]. The BSC—derived (BSC-D) represents the fraction of ultrasound energy that is scattered back from the liver parenchyma and provides an excellent AUC (0.925, std. 0.035) from three prospective studies (Siemens S3000, Siemens Healthineers, Erlangen, Germany) (Samsung RS85, Samsung Medison, Seoul, Republic of Korea) [11,43,49]. However, the backscatter-based or attenuation-based fatty liver evaluation require a reference phantom during measurement [25].

UDFF takes advantage of both AC and BSC, demonstrating very good diagnostic performance when compared to MRI-PDFF. This quantitative ultrasound technique is cost-effective and comfortable for patients, providing a direct percentage of liver fat infiltration. However, there are variations in the proposed diagnostic cut-offs of UDFF across different studies [6,14,15,21]. As the severity of steatosis increases, the reproducibility and accuracy of the method may be affected, indicating the need for further research and adjustment of the parameters [25]. Data from studies indicate that using appropriate thresholds (>5%, >10%, and >15% for mild, moderate, and severe steatosis, respectively), the UDFF method provides diagnostic performance comparable to that of MRI-PDFF [24]. Also, the variability observed under different measurement conditions highlights the necessity for standardized protocols [5,22]. Factors like body positioning, probe pressure, different transducers, and frequencies may influence the results [26]. Additionally, due to its novel technology, further training of operators is required for proper and more reproducible application of the method [26].

In this review, key contributors to between-study variability included MRI factors of field strength and UDFF protocol (ROI depth, number of measurements, probe pressure, body position, breath-hold, and fasting). Heterogeneity was moderate for correlation (I^2^~67%) and low to high for ICC depending on the domain (inter vs. intra), consistent with variations in MRI implementation (1.5T vs. 3T, thresholding at 5.0 or 5.5%), ROI comparability (segmental vs. whole-liver; depth), and patient factors (BMI/Skin-to-Capsule distance). From a clinical point of view, this review supports UDFF as a reliable, non-invasive, and cost-effective alternative to MRI-PDFF for the routine assessment of hepatic steatosis. However, it needs further investigation in terms of application features.

A recent meta-analysis provided similar results to our diagnostic performance metrics for UDFF, pooling data from nine studies with 1150 patients and reporting a pooled sensitivity of 90.4%, specificity of 83.8%, and an AUC of 0.93 when compared to MRI-PDFF as the reference standard [50]. Our review not only confirms the high diagnostic accuracy of UDFF but also offers an in-depth analysis of methodological variations across studies, including differences in transducer frequency, patient positioning, breath-hold techniques, ROI depth, and measurement count. This practical narrative is intended to translate pooled performance into reproducible workflows [18,28]. More specifically, in this study, we provide implementation-grade guidance for UDFF. By mapping official vendor guidance against study-level deviations and distilling standardized recommendations (fasting, position/respiration, ROI depth, measurement count, QC, and threshold calibration), we present a practical dataset that facilitates reproducible adoption across sites and populations for future research, as will be stated in the next sub-paragraph.

In cost-effective terms, UDFF as an ultrasound modality offers increased availability and short acquisition time, downstreaming MRI demand. From an economic point of view, ultrasound provides cost-effective equipment, less staff needed, cheaper consumables, and more repetitions per examination, and it has therefore been reported as less expensive than MRI in liver applications [51,52]. Beyond economics, ultrasound is also the most environmentally sustainable cross-sectional imaging modality, with markedly lower energy consumption and carbon footprint compared with MRI; multiple evaluations identify MRI and CT as the largest contributors to departmental imaging emissions, whereas ultrasound has the lowest per-scan footprint [53,54,55,56,57]. Thus, UDFF offers a way to lower costs and shrink the imaging carbon footprint without compromising diagnostic safety.

### 4.1. Economic and Environmental Considerations

In cost-effective terms, UDFF as an ultrasound modality offers increased availability and short acquisition time, downstreaming MRI demand. From an economic point of view, ultrasound provides cost-effective equipment, less staff needed, cheaper consumables, easy repetitions per examination, and rapid examination time. Therefore, it has been reported as less expensive than MRI in liver applications [51,52]. The weighted economic burden of MRI is reflected in examination cost differences throughout the continents. For instance, the NHS (United Kingdom) reports a typical tariff of GBP 177 (without contrast) and GBP 264 (with contrast), while ultrasound is GBP 48–61 [53]. Similarly, in the United States, MRI remains substantially more costly than US. Representative Medicare payments for MRI angiography procedures report an average range of USD 340–386 [54]. Therefore, studies should include costs for US vs. MRI; capital costs and operational costs with details regarding minutes per exam and staff needed; quality costs, including the possibility of repletion and staff education needed; and downstream costs, referring to avoided clinic visits and referrals to MRI. Practically, departments can run a 12-month budget/impact analysis comparing MRI-PDFF vs. UDFF and analyze costs in their country to further comprehend the economic benefits of US.

Beyond economics, ultrasound is also the most environmentally sustainable cross-sectional imaging modality, with markedly lower energy consumption and carbon footprint compared with MRI; multiple evaluations identify MRI and CT as the largest contributors to departmental imaging emissions, whereas ultrasound has the lowest per-scan footprint [55,56,57,58,59]. Annual MRI consumption is about ~8–170 MWh, with 72–91% nonproductive energy and significant power consumption even in “system off” states [57,58,59]. All assessments rank ultrasound as the least environmentally impactful modality [55,56,57,58,59]. Thus, UDFF offers a way to lower costs and shrink the imaging carbon footprint without compromising diagnostic safety.

### 4.2. Future Research

Future research should prioritize multi-center trials across diverse geographic regions and patient groups, including children and individuals with advanced liver disease. Longitudinal studies are also needed to clarify the role of UDFF in disease monitoring and treatment response. Future studies should also minimize heterogeneity by adopting harmonized MRI-PDFF protocols. More specifically, based on this review, we recommend 4 to 6 h of fasting where feasible, default to supine, right arm overhead, end-expiratory breath-hold, and minimal probe pressure with detailed documentation regarding transducer, frequency, and presets. For site acquisition, use 1.5 to 2.0 cm as the standard. If StC distance is large, consider lower frequency and keep ROI within a fixed depth band (report StC distance and ROI depth). Acquire at least 5 to 10 valid measurements. Finally, calibrate UDFF cut-offs to your MRI-PDFF threshold and population and state the MRI-PDFF threshold used.

Studies should also report grade-specific AUC, Se/Sp, r, ICC, and Bland–Altman agreement. Multi-center, multi-vendor trials should explicitly evaluate the impact of MRI threshold, ROI depth, and adiposity (BMI/visceral fat) on S1 performance.

Standardized comparisons of UDFF with other quantitative ultrasound techniques and MRI-PDFF will be critical to establishing its role in routine clinical practice.

### 4.3. Strengths and Limitations

A major strength of this review is the detailed comparative analysis of UDFF diagnostic accuracy with MRI-PDFF as the non-invasive gold standard. Also, it provides practical insights into procedural adjustments and their rationales. By incorporating studies that investigate potential confounders (BMI, probe pressure, fasting state, and ROI placement), this review addresses critical methodological concerns influencing UDFF accuracy and reproducibility. The limitation lies in the variability of studies in terms of patient populations, device settings, and measurement protocols. While not all parameters were uniformly reported, the inclusion of reproducibility metrics and threshold variability offers valuable practical context for UDFF implementation, complementing core accuracy findings. Another limitation of this review is that the initial search was restricted to PubMed and Scopus. To address this concern, we expanded the search to include Embase, Web of Science, and the Cochrane Library, which did not yield additional eligible studies. Finaly, UDFF, as evaluated in the current evidence base, is proprietary to a single vendor. Therefore, diagnostic performance estimates may not generalize to other quantitative ultrasound fat fraction implementations (e.g., UGAP, TAI/TSI, iATT, USFF). Recent work has demonstrated significant inter-platform variability in ultrasound-based fat quantification [18]. Until multi-vendor cross-calibration and harmonized protocols are established, we recommend caution toward direct numerical equivalence across platforms.

## Figures and Tables

**Table 1:** UDFF diagnostic performance in comparison to MRI-PDFF.

Authors (Year)	Sample Size	MRI–PDFF Vendor/ Threshold (%)	Cofactors Evaluated	UDFF/ MRI-PDFF Correlation	Intra-Observer Agreement UDFF	Inter-Observer Agreement UDFF	UDFF AUC (Diagnostic Cut-Off > 5%)
Labyed et al. (2020) [11]	101 adults with suspected or known NAFLD	3T MRI (Signa Excite HD, GE Healthcare)/≥5.0%	BMI	r: 0.870	NR	NR	0.97
Dillman et al. (2022) [13]	56 overweight and obese adults	3T MRI (GE SIGNA Architect)/≥5.5%	BMI, waist circumference, body positioning	ICC: 0.840 r: 0.820	0.98	NR	0.90 for ≥S1
De Robertis et al. (2023) [3]	122 healthy adults (steatosis grade < 2)	3T MRI (Philips Ingenia Elition S)/≥5.0%	NR	r: 0.808	NR	NR	0.747 for ≥S1
Kubale et al. (2024) [14]	187 patients undergoing liver MRI for various indications	3T MRI/≥5.0%	Dietary state	ICC: 0.790	0.985	0.935	0.90 for ≥S1, 0.95 for ≥S2, 0.95 for ≥S3
Qi et al. (2024) [6]	176 patients undergoing liver evaluation	3T MRI (Siemens Magnetom Verio)/≥5.5%	ΒΜΙ	ICC: 0.899 r: 0.831	0.992	0.951	0.85 for ≥S1, 0.95 for ≥S2, 0.95 for ≥S3
Song et al. (2024) [22]	105 MASLD patients	1.5T MRI (Siemens Magnetom Aera) on 25 patients (25/105)/≥5.0%	BMI, Skin-to-Capsule (StC) distance, body position, respiration, dietary state	NR	0.960	0.940	NR
Wang et al. (2024) [15]	115 MASLD patients	3T MRI-PDFF	Age, BMI, waist-to-hip ratio/ ≥5.0%	r: 0.910	NR	0.960	0.99 for ≥S1, 0.96 for ≥S2, 0.97 for ≥S3
Total of 7 prospective studies on UDFF	862 patients with MASLD or risk factors for developing MASLD	Various MRI systems (mostly 3T)	Almost the same in each study	ICC (average) = 0.843 (std. 0.04) r (average) = 0.848 (std. 0.04)	ICC (average) = 0.98	ICC (average) = 0.978	AUC (average) for ≥S1: 0.887)

NR = not reported in the source.

**Table 2:** UDFF diagnostic performance in comparison to other modalities.

Authors (Year)	Sample Size	Comparisons with Other Modalities	Cofactors Evaluated	UDFF Correlations	Intra-Observer Agreement	Inter-Observer Agreement	UDFF AUC (Diagnostic Cut-Off > 5%)
Gao et al. (2021) [23]	21 adult volunteers	Auto-pSWE, pSWE	BMI, StC distance	NR	0.97–0.99	0.87–0.96	NR
Sporea et al. (2022) [24]	271 patients, with or without chronic liver disease	CAP	BMI	UDFF/CAP r: 0.750	NR	NR	0.92 for ≥S1, 0.95 for ≥S2, 0.93 for ≥S3
Huang et al. (2024) [25]	38 adults with suspected MASLD	B-mode ultrasound	Age, gender, hepatic segment, StC distance	NR	0.882	NR	NR
Tavaglione et al. (2024) [26]	302 obese individuals at high risk for MASLD	CAP and Hamaguchi scores	BMI, ALT, triglycerides, visceral adipose tissue	UDFF/CAP r: 0.730 UDFF/Hamaguchi score r: 0.790	NR	NR	0.92 for ≥S1
Chen et al. (2024) [1]	6 Bama minipigs	Histopathological biopsy	BMI, triglycerides, total cholesterol, HDL, LDL	UDFF/NAFLD Activity Score r: 0.800	NR	NR	0.95 for ≥S1
Jeon et al. (2024) [18]	41 adults with suspected MASLD	USFF	Visual hepatic steatosis grade, BMI, StC distance	UDFF/USFF r: 0.748 ICC: 0.842	NR	0.963	NR
Nakamura et al. (2025) [21]	73 MASLD patients	Liver biopsy	BMI, StC distance	NR	NR	NR	0.956 for ≥S1, 0.926 for ≥S2, 0.971 for ≥S3
Meng et al. (2025) [27]	124 obese PCOS patients	Shear Wave Velocity (SWV), MAFLD stage	BMI, insulin resistance (HOMA-IR), testosterone, lipid profile	UDFF/MAFLD r: 0.603	NR	NR	0.935 for ≥S1

NR = not reported in the source.

**Table 3:** UDFF sensitivity and specificity at different cut-off values.

Authors (Year)	UDFF Mild Steatosis Cut-Off (%) (Se/Sp, AUC)	UDFF Moderate Steatosis Cut-Off (%) (Se/Sp, AUC)	UDFF Severe Steatosis Cut-Off (%) (Se/Sp, AUC)
Labyed et al. (2020) [11]	5.0 (NR/NR, 0.95)	10.0 (NR/NR, 0.95)	NR
Sporea et al. (2022) [24]	5.0	10.0	15.0
Dillman et al. (2022) [13]	5.5 (0.94/0.64, 0.90)	NR	NR
De Robertis et al. (2023) [3]	5.0 (0.80/0.66, 0.75)	NR	NR
Chen et al. (2024) [1]	5.5 (0.80/0.96, 0.95)	NR	NR
Qi et al. (2024) [6]	5.5 (0.79/0.82, 0.85)	15.5 (0.86/0.91, 0.95)	17.5 (0.89/0.90, 0.95)
Kubale et al. (2024) [14]	6.5 (NR/NR, 0.90)	17.4 (NR/NR, 0.95)	22.1 (NR/NR, 0.95)
Wang et al. (2024) [15]	6.0 (NR/NR, 0.99)	15.0 (NR/NR, 0.96)	23.0 (NR/NR, 0.97)
Meng et al. (2025) [27]	4.5 (0.92/0.85, 0.94)	NR	NR
Nakamura et al. (2025) [21]	6.0 (0.95/0.82, 0.96)	13.0 (0.77/0.88, 0.93)	23.0 (1.00/0.94, 0.97)
Recommendation for further study	5–6% UDFF for initial MASLD detection/mild steatosis	10–15% for mild to moderate and 15–17.5% for moderate steatosis	17.5–22% for moderate to severe steatosis and 23% for severe steatosis

Se/Sp: sensitivity/specificity; AUC: Area Under the Curve; NR: not reported in the source.

## Data Availability

No new data were created.

## Abbreviations

The following abbreviations are used in this manuscript:

AC	Attenuation coefficient
AUC	Area Under the Curve
BSC	Backscatter coefficient
BSC-D	Backscatter coefficient—derived
CAP	Controlled Attenuation Parameter
HOMA-IR	Homeostatic Model Assessment of Insulin Resistance
iATT	Integrated Attenuation
MAFLD	Metabolic-dysfunction-associated fatty liver disease
MASLD	Metabolic-dysfunction-associated steatotic liver disease
MRI	Magnetic resonance imaging
MRI-PDFF	Magnetic Resonance Imaging Proton Density Fat Fraction
NAFLD	Nonalcoholic fatty liver disease
pSWE	point SWE
QUS	Quantitative ultrasound
ROI	Region of Interest
StC	Skin-to-Capsule distance
SWE	Shear Wave Elastography
SWV	Shear Wave Velocity
TAI	Tissue Attenuation Imaging
TSI	Tissue Scatter Distribution Imaging
UDFF	Ultrasound-Derived Fat Fraction
UGAP	Ultrasound-Guided Attenuation Parameter
USFF	Ultrasound fat fraction

## Appendix A

**Table A1:** QUADAS-2 Assessment.

Study (Author, Year)	Patient Selection	Index Test (UDFF)	Reference Standard (MRI-PDFF)	Flow and Timing	Overall Risk of Bias
Labyed et al., 2020 [11]	Low (adults with NAFLD, clear inclusion)	Low (UDFF applied consistently, blinding not specified → could be unclear)	Low (3T MRI-PDFF, robust method)	Low	Low–moderate
Dillman et al., 2022 [13]	Low (overweight/obese adults prospectively enrolled)	Low (prespecified UDFF measurements, reproducibility reported)	Low (3T MRI-PDFF, reference standard)	Low	Low
De Robertis et al., 2023 [3]	Low (healthy adults, clear criteria)	Unclear (thresholds less well-defined, reproducibility not fully reported)	Low	Low	Low–moderate
Kubale et al., 2024 [14]	Low (broad patient population)	Unclear (variability with dietary state, thresholds not prespecified)	Low (3T MRI-PDFF)	Low	Low–moderate
Qi et al., 2024 [6]	Low (well-defined adult cohort)	Low (multiple cofactors assessed, thresholds prespecified)	Low (3T MRI-PDFF, Siemens)	Low	Low
Song et al., 2024 [22]	Low (MASLD patients)	Low (careful analysis of body position/respiration, reproducibility tested)	Low (MRI-PDFF, subset at 1.5T)	Low	Low
Wang et al., 2024 [15]	Low (MASLD patients, risk-stratified)	Low (reproducibility high, thresholds prespecified)	Low (3T MRI-PDFF)	Low	Low

Index Test: UDFF interpretation was blinded to MRI results → could be unclear. Patient Selection: All were prospective, with relatively clear inclusion → low. Reference Standard: MRI-PDFF → low. Flow and Timing: Imaging in same session → low.
